# Supplementary material for: Thalamocortical axons control the cytoarchitecture of neocortical layers by area-specific supply of VGF
Source: eLife. 2022 Mar 15;11:e67549. doi: 10.7554/eLife.67549 (PMC8959604; doi:10.7554/eLife.67549)
Supplement: Transparent reporting form [file elife-67549-transrepform1.docx]

***eLife’s* transparent reporting form**

We encourage authors to provide detailed information *within their submission* to facilitate the interpretation and replication of experiments. Authors can upload supporting documentation to indicate the use of appropriate reporting guidelines for health-related research (see [EQUATOR Network](http://www.equator-network.org/%20)), life science research (see the [BioSharing Information Resource](https://biosharing.org/)), or the [ARRIVE guidelines](http://www.plosbiology.org/article/info:doi/10.1371/journal.pbio.1000412) for reporting work involving animal research. Where applicable, authors should refer to any relevant reporting standards documents in this form.

If you have any questions, please consult our Journal Policies and/or contact us: [editorial@elifesciences.org](mailto:editorial@elifesciences.org).

**Sample-size estimation**

- You should state whether an appropriate sample size was computed when the study was being designed
- You should state the statistical method of sample size computation and any required assumptions
- If no explicit power analysis was used, you should describe how you decided what sample (replicate) size (number) to use

Please outline where this information can be found within the submission (e.g., sections or figure legends), or explain why this information doesn’t apply to your submission:

No sample size was computed. We have used a minimum of 3 animals per analysis and repeated the experiments for at least of 3 animals with more than 3 independent sections analyzed, except for those shown in Figure 4C (N=2 mice) and Figure 2-figure supplement 2 (P1 TCA-ablated, N=2 mice). The actual number of animals and independent sections is indicated in the results and figure legends sections.

**Replicates**

- You should report how often each experiment was performed
- You should include a definition of biological versus technical replication
- The data obtained should be provided and sufficient information should be provided to indicate the number of independent biological and/or technical replicates
- If you encountered any outliers, you should describe how these were handled
- Criteria for exclusion/inclusion of data should be clearly stated
- High-throughput sequence data should be uploaded before submission, with a private link for reviewers provided (these are available from both GEO and ArrayExpress)

Please outline where this information can be found within the submission (e.g., sections or figure legends), or explain why this information doesn’t apply to your submission:

We have examined a minimum of 3 animals per analysis and repeated the experiments for at least of 3 animals with more than 3 independent sections analyzed, except for those shown in Figure 4C (N=2 mice) and Figure 2-figure supplement 2 (P1 TCA-ablated, N=2 mice) because of discontinued reagents and shortage of animal resources, respectively. The actual number of animals and independent sections is indicated in Results and Figure legends.

**Statistical reporting**

- Statistical analysis methods should be described and justified
- Raw data should be presented in figures whenever informative to do so (typically when N per group is less than 10)
- For each experiment, you should identify the statistical tests used, exact values of N, definitions of center, methods of multiple test correction, and dispersion and precision measures (e.g., mean, median, SD, SEM, confidence intervals; and, for the major substantive results, a measure of effect size (e.g., Pearson's r, Cohen's d)
- Report exact p-values wherever possible alongside the summary statistics and 95% confidence intervals. These should be reported for all key questions and not only when the p-value is less than 0.05.

Please outline where this information can be found within the submission (e.g., sections or figure legends), or explain why this information doesn’t apply to your submission:

Methods for statistical analyses are described in the materials and methods section. N values, mean, SEM and exact P-values are indicated in the figure legends. We used Mann-Whitney U test as a non-parametric statistical evaluation for cases with less than 30 data points. The P-value less than 0.05 was considered to be statistically significant.

(For large datasets, or papers with a very large number of statistical tests, you may upload a single table file with tests, Ns, etc., with reference to sections in the manuscript.)

**Group allocation**

- Indicate how samples were allocated into experimental groups (in the case of clinical studies, please specify allocation to treatment method); if randomization was used, please also state if restricted randomization was applied
- Indicate if masking was used during group allocation, data collection and/or data analysis

Please outline where this information can be found within the submission (e.g., sections or figure legends), or explain why this information doesn’t apply to your submission:

Samples were allocated to control or mutant groups upon genotyping, or to control that does not carry 5HTT-Cre allele or TCA-ablated groups with 5HTT-Cre allele, both of which diphtheria toxin administrated. This information can be found in the results, figure legends, and materials and methods section.

**Additional data files (“source data”)**

- We encourage you to upload relevant additional data files, such as numerical data that are represented as a graph in a figure, or as a summary table
- Where provided, these should be in the most useful format, and they can be uploaded as “Source data” files linked to a main figure or table
- Include model definition files including the full list of parameters used
- Include code used for data analysis (e.g., R, MatLab)
- Avoid stating that data files are “available upon request”

Please indicate the figures or tables for which source data files have been provided:

Data files for quantitative and statistical analyses were uploaded for Figures 1E, 2C, 2D, 2F, 3D, 4C, 5D, 3C, 3F, 4D, 4E, 5D, 6C, 7C, 7D, 7G, 7I, 7K, Figure 1-figure supplement 1C, Figure 2-figure supplement 1C, Figure 2-figure supplement 2B, 2D, Figure 5-figure supplement 2C, Figure 7-figure supplement 1B, 1D, 1F, 1H, and Figure 7-figure supplement 2B.
